# Temporal trend of acute myocardial infarction-related mortality and associated racial/ethnic disparities during the omicron outbreak

**DOI:** 10.2478/jtim-2023-0125

**Published:** 2023-12-20

**Authors:** Yee Hui Yeo, Yue Zhang, Xinyuan He, Fan Lv, Jignesh K. Patel, Fanpu Ji, Susan Cheng

**Affiliations:** Karsh Division of Gastroenterology and Hepatology, Cedars-Sinai Medical Center, Los Angeles, California, USA; Department of Infectious Disease, The Second Affiliated Hospital of Xi’an Jiaotong University, Xi'an, Shaanxi Province, China; School of Mathematics and Statistics, Xi’an Jiaotong University, Xi'an, Shaanxi Province, China; Department of Cardiology, Smidt Heart Institute, Cedars-Sinai Medical Center, Los Angeles, California, USA; National & Local Joint Engineering Research Center of Biodiagnosis and Biotherapy, the Second Affiliated Hospital of Xi’an Jiaotong University, Xi’an, Shaanxi Province, China; Shaanxi Provincial Clinical Medical Research Center of Infectious Diseases, Xi'an, Shaanxi Province, China; Key Laboratory of Surgical Critical Care and Life Support (Xi'an Jiaotong University), Ministry of Education, Xi’an, Shaanxi Province, China

Prior studies have reported a surge of acute myocardial infarction (AMI)-related deaths and exacerbating racial/ethnic disparities during the first two years of the COVID-19 pandemic.^[1, 2, 3]^ The emergence of different variants, complicated by the evolving healthcare landscape and seasonal changes, impose distinct impacts on the cardiovascular disease burden of the population.^[4, 5, 6]^ However, little is known about the recent trend of AMI-related mortality during the outbreaks of Omicron variants and the temporal trend for the burden of cardiovascular mortality among different racial/ethnic groups.

Mortality data were obtained from the CDC’s National Vitals Statistics System (NVSS) dataset, which registered death certificates covering over 99% of decedents with a periodical update.^[7]^ The study analyzed AMI-related deaths occurring between 1/1/2018 and 9/30/2022, encompassing the surge periods of COVID-19 dominated by the Alpha, Delta, and Omicron variants. The age-standardized mortality rate (ASMR) for AMI-related deaths was calculated for both the pre-pandemic and pandemic epochs and categorized by dominant strains and racial/ethnic groups. Predicted ASMRs were estimated according to the pre-pandemic trend. Joinpoint analysis was performed to determine the monthly percentage change in AMI-related mortality and to compare the same periods in 2021–2022.

Monthly data of 722, 472 AMI-related deaths between January 2018 and September 2022 was shown. From March 2020, the fluctuation in AMI-related death rate, stratified by the predominant variant and COVID-19 status, is shown in Figure 1A. The rise in AMI-related mortality was in temporal association with each variant outbreak, and COVID-19 was the main reason for the surge. Notably, the Omicron outbreak in January 2022 had the highest rise in AMI-related death rate, surpassing the prior Delta and Alpha outbreaks, followed by a significant decrease from January to September 2022. While there was no significant difference in the decreasing trends between January-June 2021 and January-June 2022 (Tab. S1), there were significant differences in the trend between June-September 2021 and June-September 2022 (Tab. S2) (*P* < 0.05).

**Figure 1:**
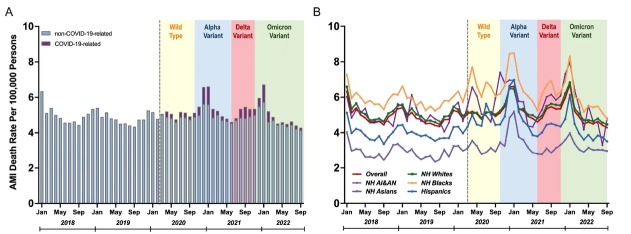
Temporal Trends and Seasonality of Acute Myocardial Infarction-related Deaths with and without associated COVID-19 (A), by Race/ethnicity (B). COVID-19 associated death was defined as AMI-related death with COVID-19 listed as one of the conditions.

The temporal association between outbreaks and AMI-related mortality varied across all racial/ethnic groups. The highest absolute ASMRs and most pronounced augmentation in mortality were observed in non-Hispanic Blacks and non-Hispanic American Indians/Alaska Natives (AI/AN), followed by non-Hispanic Whites (Figure 1B). However, when compared to predicted ASMRs, the ASMRs of most racial/ethnic groups in September 2022 were close to or lower than the predicted values, except for non-Hispanic Asians (Figure 2). Between January-June 2022, non-Hispanic AI/AN showed the most significant decrease in AMI-related mortality-9.40% (95%CI, -18.4%–0.5%), followed by Hispanic and non-Hispanic Whites. Most racial/ethnic groups demonstrated significant differences in the trend between June-September 2021 and June-September 2022 (all *P* < 0.05), except for non-Hispanic AI/AN due to the fluctuation during this period and non-Hispanic Asians.

**Figure 2:**
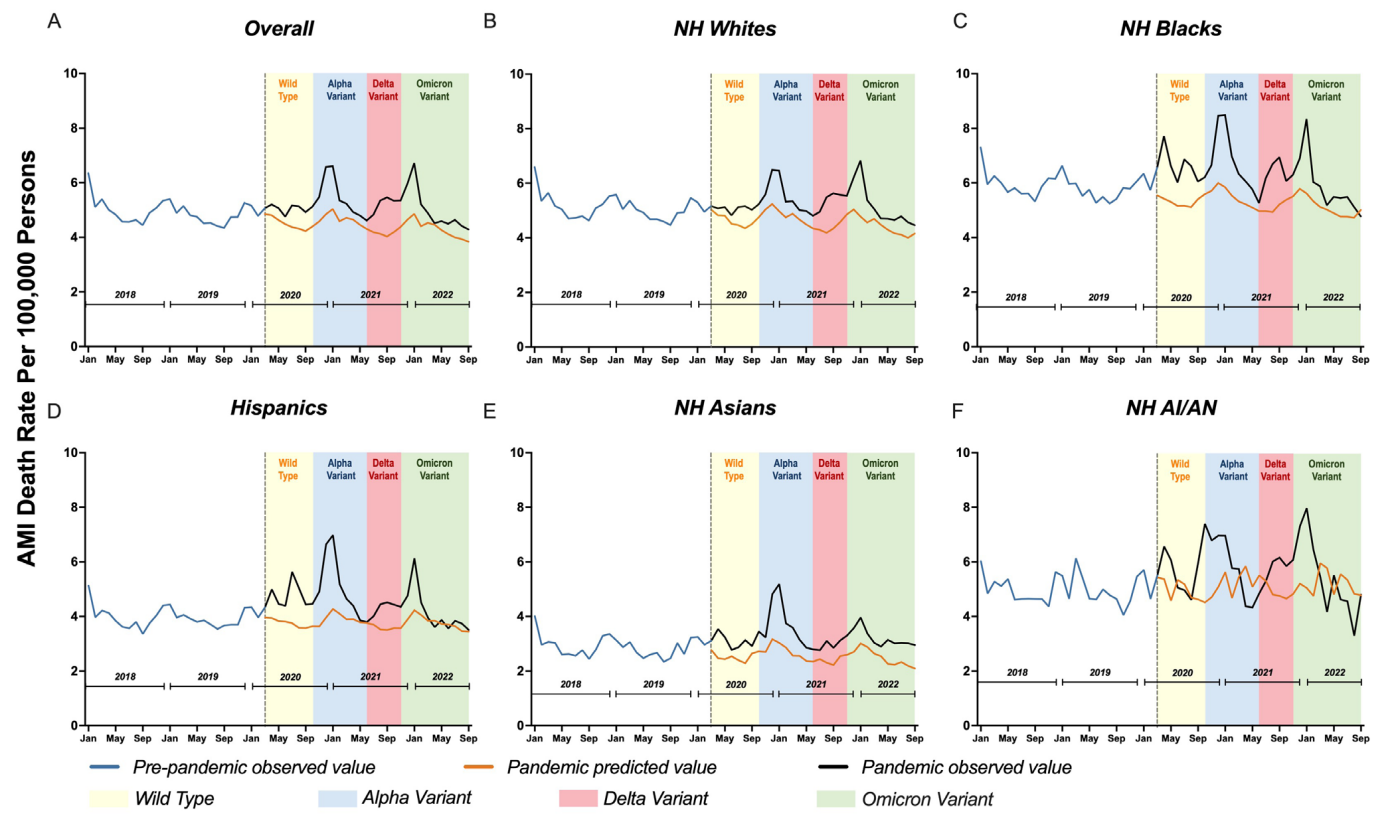
Monthly Mortality Rate of Acute Myocardial Infarction-related Deaths by Race/ethnicity. Data spanned from 1/1/2018 to 9/30/2022.

Our findings demonstrated a disparity in AMI-related mortality across racial/ethnic groups and showed a recovery of AMI-related mortality in the U. S. from January-September 2022. Although non-Hispanic Blacks and AI/AN were most affected, they showed lower than predicted ASMRs in September 2022.

Prior studies have reported pandemic-associated increases in cardiovascular morbidity, particularly in traditionally higher-risk populations such as non-Hispanic Blacks.^[8,9]^ Emerging data suggest that even mild COVID-19 can activate inflammatory or metabolic stress responses that may exacerbate pre-existing cardiovascular risk.^[10, 11, 12]^ Efforts have been made to transform the interrupted healthcare system to address health equity. Our analyses have several limitations. Firstly, we were limited to the use of ICD-10 codes in defining AMI-related mortality may lead to misclassification bias. Secondly, As the data of CDC Wonder dataset are derived from the registry of death certificates, it only provides the information related to acute myocardial infarction. Therefore, we are unable to provide information regarding morbidity. Lastly, we did not use statistical methods to compare disparity of ASMRs in AMI-related mortality across racial/ethnic groups. Further studies with adjudicated cases are needed to validate the findings.

Our results presented the temporal trend of AMI-related mortality before and throughout the pandemic and pointed to a racial/ethnic disparity. Despite decreased mortality across all subgroups during the Omicron outbreak, further inventions are needed to address the inequity in cardiovascular disease burden.

## Supplementary Material

Supplementary material

## References

[1] Shiels MS, Haque AT, Haozous EA, Albert PS, Almeida JS, García-Closas M (2021). Racial and Ethnic Disparities in Excess Deaths During the COVID-19 Pandemic, March to December 2020. Ann Intern Med.

[2] Li J, Sun L, Wang F, Liu B, Li H, Tang G (2021). Relation between Cardiac Injury and Elevated Levels of Inflammatory Biomarkers in Patients with Severe COVID-19. Cardiovasc Innov Appl.

[3] Yeo YH, Wang M, He X, Lv F, Zhang Y, Zu J (2023). Excess risk for acute myocardial infarction mortality during the COVID-19 pandemic. J Med Virol.

[4] Skow RJ, Nandadeva D, Grotle AK, Stephens BY, Wright AN, Fadel PJ (2022). Impact of breakthrough COVID-19 cases during the omicron wave on vascular health and cardiac autonomic function in young adults. Am J Physiol Heart Circ Physiol.

[5] Zhu Z, Tang J, Chai X, Fang Z, Hu Q, Hu X (2021). Similarities and Differences of CT Features between COVID- 19 Pneumonia and Heart Failure. Cardiovasc Innov Appl.

[6] Liu H, Wang S, Yang S, Luo SX, Jie J, Hua S (2023). Characteristics of the Severe Acute Respiratory Syndrome Coronavirus 2 Omicron BA.2 Subvariant in Jilin, China from March to May 2022. J Transl Int Med.

[7] Prevention TCfDCa (2022). CDC WONDER.

[8] Wadhera RK, Figueroa JF, Rodriguez F, Liu M, Tian W, Kazi DS (2021). Racial and Ethnic Disparities in Heart and Cerebrovascular Disease Deaths During the COVID-19 Pandemic in the United States. Circulation.

[9] Wadhera RK, Shen C, Gondi S, Chen S, Kazi DS, Yeh RW (2021). Cardiovascular Deaths During the COVID-19 Pandemic in the United States. J Am Coll Cardiol.

[10] Xiong TY, Redwood S, Prendergast B, Chen M (2020). Coronaviruses and the cardiovascular system: acute and long-term implications. Eur Heart J.

[11] Murata F, Maeda M, Ishiguro C, Fukuda H (2022). Acute and delayed psychiatric sequelae among patients hospitalised with COVID-19: a cohort study using LIFE study data. Gen Psychiatr.

[12] Li C, Wang DW, Zhao C (2021). Cardiovascular Involvement in Patients with 2019 Novel Coronavirus Disease. J Transl Int Med.

