# Supplementary material for: Temporal trend of acute myocardial infarction-related mortality and associated racial/ethnic disparities during the omicron outbreak
Source: J Transl Int Med. 2023 Dec 20;11(4):468–70. doi: 10.2478/jtim-2023-0125 (PMC10732487; doi:10.2478/jtim-2023-0125)
Supplement: Supplementary file 1 — Supplementary material [file jtim-2023-0125_sm.pdf]

Supplementary Table 1: Comparison of Trends for Acute Myocardial Infarction-related Deaths between 1/1/2021-6/30/2021 and 1/1/2022-6/30/2022, by race/ethnicity

Overall

Year	APC	*P* value
2021	-6.04* [-9.6 to-2.3]	0.011
2022	-6.81* [-12.1 to-1.2]	0.029
Comparison		
2022 (referent)	0.8 [-3.9 to 5.4]	1

Hispanics

Year	APC	*P* value
2021	-10.76* [-15.6 to-5.6]	0.005
2022	-8.91* [-15.8 to-1.4]	0.030
Comparison		
2022 (referent)	-1.9 [-8.0 to 4.3]	1

NH-whites

Year	APC	*P* value
2021	-4.85* [-8.3 to-1.3]	0.020
2022	-6.62* [-11.5 to-1.4]	0.024
Comparison		
2022 (referent)	1.8 [-2.6 to 6.1]	<0.1

NH-blacks

Year	APC	*P* value
2021	-8.21* [-11.3 to-5.0]	0.002
2022	-7.00 [-14.0 to 0.6]	0.061

Comparison		
2022 (referent)	-1.2 [-6.8 to 4.4]	1

NH-Asians

**Year**	**APC**	***P* value**
2021	-10.88* [-15.8 to-5.6]	0.005
2022	-4.54 [-9.6 to 0.8]	0.078
Comparison		
2022 (referent)	-6.3* [-11.5 to-1.2]	<0.1

NH-AI/AN

**Year**	**APC**	***P* value**
2021	-8.07* [-14.6 to-1.0]	0.034
2022	-9.40 [-18.4 to 0.5]	0.058
Comparison		
2022 (referent)	1.3 [-6.9 to 9.5]	1

Supplementary Table 2: Comparison of Trends for Acute Myocardial Infarction-related Deaths between 6/30/2021-9/30/2021 and 6/30/2022-9/30/2022, by race/ethnicity

Overall

**Year**	**APC**	***P* value**
2021	6.27* [1.6 to 11.2]	0.029
2022	-1.88 [-6.8 to 3.3]	0.253
Comparison		
2022 (referent)	8.2* [5.0 to 11.3]	<0.1

Hispanics

Year	APC	*P* value
2021	6.44* [1.2 to 11.9]	0.033
2022	-0.71 [-10.0 to 9.5]	0.783
Comparison		
2022 (referent)	7.1* [2.1 to 12.2]	<0.1

NH-whites

Year	APC	*P* value
2021	5.93* [1.2 to 10.9]	0.033
2022	-1.70 [-6.2 to 3.0]	0.257
Comparison		
2022 (referent)	7.6* [4.6 to 10.7]	<0.1

NH-blacks

Year	APC	*P* value
2021	9.51* [0.6 to 19.2]	0.044
2022	-4.55 [-10.0 to 1.2]	0.077
Comparison		
2022 (referent)	14.1* [9.1 to 19.0]	<0.1

NH-Asians

Year	APC	*P* value
2021	1.75 [-8.9 to 13.7]	0.570
2022	-0.66 [-2.5 to 1.2]	0.262
Comparison		
2022 (referent)	2.4 [-2.8 to 7.6]	<0.1

NH-AI/AN

Year	APC	*P* value
2021	8.83* [2.3 to 15.8]	0.028
2022	-2.10 [-34.8 to 46.9]	0.843
Comparison		
2022 (referent)	10.9 [-7.4 to 29.3]	<0.1
